# A Highly Accurate Inclusive Cancer Screening Test Using *Caenorhabditis elegans* Scent Detection

**DOI:** 10.1371/journal.pone.0118699

**Published:** 2015-03-11

**Authors:** Takaaki Hirotsu, Hideto Sonoda, Takayuki Uozumi, Yoshiaki Shinden, Koshi Mimori, Yoshihiko Maehara, Naoko Ueda, Masayuki Hamakawa

**Affiliations:** 1 Graduate School of Systems Life Sciences, Kyushu University, Fukuoka, Japan; 2 Department of Biology, Graduate School of Sciences, Kyushu University, Fukuoka, Japan; 3 Division of Applied Medical Sensing, Research and Development Center for Taste and Odor Sensing, Kyushu University, Fukuoka, Japan; 4 Department of Biology, Faculty of Science, Kyushu University, Fukuoka, Japan; 5 Department of General Surgery, Imari-Arita Kyoritsu Hospital, Saga, Japan; 6 Department of Surgery, Kyushu University Beppu Hospital, Oita, Japan; 7 Department of Surgery and Science, Graduate School of Medical Sciences, Kyushu University, Fukuoka, Japan; East Carolina University, UNITED STATES

## Abstract

Early detection and treatment are of vital importance to the successful eradication of various cancers, and development of economical and non-invasive novel cancer screening systems is critical. Previous reports using canine scent detection demonstrated the existence of cancer-specific odours. However, it is difficult to introduce canine scent recognition into clinical practice because of the need to maintain accuracy. In this study, we developed a Nematode Scent Detection Test (NSDT) using *Caenorhabditis elegans* to provide a novel highly accurate cancer detection system that is economical, painless, rapid and convenient. We demonstrated wild-type *C*. *elegans* displayed attractive chemotaxis towards human cancer cell secretions, cancer tissues and urine from cancer patients but avoided control urine; in parallel, the response of the olfactory neurons of *C*. *elegans* to the urine from cancer patients was significantly stronger than to control urine. In contrast, G protein α mutants and olfactory neurons-ablated animals were not attracted to cancer patient urine, suggesting that *C*. *elegans* senses odours in urine. We tested 242 samples to measure the performance of the NSDT, and found the sensitivity was 95.8%; this is markedly higher than that of other existing tumour markers. Furthermore, the specificity was 95.0%. Importantly, this test was able to diagnose various cancer types tested at the early stage (stage 0 or 1). To conclude, *C*. *elegans* scent-based analyses might provide a new strategy to detect and study disease-associated scents.

## Introduction

Cancer is a leading cause of death worldwide, and accounted for 7.6 million deaths (around 13% of all deaths) in 2008; this figure is projected to continue rising, with an estimated 17 million deaths in 2030 [1]. Few symptoms are evident in early-stage cancer and cancer does not rapidly advance without passing through an early stage. Cancer is far more difficult to treat as it progresses to an advanced stage and symptoms become apparent. Thus, there is an urgent need for the development of a novel economical and non-invasive screening method that is able to detect early cancer. It has been reported that cancers exude odours that can be detected with high accuracy by dogs [2–5] or mice [6]. However, the practicality of introducing canine scent detection into clinical practice is difficult because its accuracy is influenced by the dogs’ concentration. Lower organisms that can detect odours emitted by cancers with the high accuracy of dogs may be suitable for the development of a technology to detect scents emitted by cancers. Nematodes such as *Anisakis simplex* may cause gastrointestinal anisakidosis when humans ingest contaminated raw or undercooked fish, and reports of about 30 patients with early-stage gastric cancer with anisakis larvae attached to their tumours are particularly provocative (case report and review by Sonoda, et al., 2014 [7]). Because the genome of *Caenorhabditis elegans* encodes at least 1,500 predicted G-protein-coupled receptors (GPCRs), including olfactory receptors [8,9], the aim of the present study was to use *C*. *elegans* to develop a system for detecting odorants emitted by cancers. We call this the “Nematode Scent Detection Test” (NSDT).

## Materials and Methods

### Preparation of culture medium from human cancer cells and fibroblasts

The human cancer cell lines SW480, COLO201, COLO205 and the nontumorigenic immortal fibroblast human cell line KMST-6 were obtained from the Japanese Collection of Research Bioresources Cell Bank (Tokyo, http://cellbank.nibio.go.jp). The human fibroblast cell line CCD-112CoN, which was derived from normal colon tissue, was acquired from the American Type Culture Collection (Manassas, VA, USA), and the other cell lines described below were from the Cell Resource Centre for Biomedical Research, Institute of Development, Aging and Cancer (Tohoku University, Sendai, Japan). All cancer cell lines were maintained at subconfluency in RPMI 1640, KMST-6 cells were cultured in MEM, and CCD-112CoN cells were cultured in EMEM. All culture media were supplemented with 10% foetal bovine serum, and the cells were maintained at 37°C in a humidified atmosphere containing 5% humidified CO_2_. The clear layers at the top of the medium were obtained and spotted onto assay plates to determine whether they induced a chemotactic response by *C*. *elegans*.

### Cancer and normal tissue sampling

Fresh surgical specimens were obtained from patients with colorectal or gastric cancer and maintained in 10 ml saline at -20°C. The normal tissue was extracted from the portion that was separated from cancer as much as possible in the resected organ. The patients had undergone surgery at the Imari Arita Kyoritsu Hospital (Arita, Japan) from January to May 2014.

### Patient and control sample donors

The participants from Imari-Arita Kyoritsu Hospital were enrolled from 12 October 2011 to 4 April 2012 for extended analyses and from 20 September 2012 to 22 May 2013 for limited analyses. There were no restrictions on meals or activities for sampling. The participants were required to be >20 years old. The participants completed a questionnaire regarding the factors that could influence the volatile molecules in their urine or serum samples including age, physical symptoms (e.g. appetite, weariness, headache, chest or abdominal distention, cough, bloody faeces, constipation, diarrhoea), pregnancy, history of cancer treatment, current use of medicine, alcohol consumption (3 or more days per week) and smoking within the previous 2 weeks. Exclusion criteria included participants who had undergone cancer surgery within the previous year, those who were not examined for cancer recurrence despite having undergone cancer surgery more than 5 years previously and those currently receiving chemotherapy. As we suspected that chemotherapy treatment or operation would change the urine chemicals in cancer patients, we sought patients who had not yet undergone any treatment. A serial number was written on each sample tube at the time of collection to identify individual information.

### Ethics Approval

This study was conducted with the approval of the institutional review boards at Imari-Arita Kyoritsu Hospital, and all subjects provided written informed consent.

### Blood serum and urine sampling

Each serum samples were separated from approximately 7 ml blood. Then, 1 to 5 ml of serum was contained in a 10 ml polypropylene screw cap tube and stored at -20°C until presentation to the test.

Approximately 10 ml urine was collected from each participant, added to a 10 ml polypropylene screw cap tube and stored at -20°C until testing. Only one sample was collected from each participant.

### Determination of tumour markers

Tumour marker concentrations were determined at Central Laboratory CRC Inc. in Fukuoka. The serum CEA concentrations were determined using chemiluminescent enzyme immunoassay [10], and the cut-off value for serum CEA was 5.0 ng/ml. The serum anti-p53 Ab concentrations were determined by enzyme immunoassay [11], with a cut-off value for serum anti-p53 Ab of 1.30 U/ml. The urinary DiAcSpm concentrations were determined using an Auto DiAcSpm reagent kit (Alfresa Pharma Co., Osaka, Japan). This assay is based on the specific binding between a bovine serum albumin-acetylspermine conjugate, as a DiAcSpm mimic, and a stable red-purple solution of colloidal gold antibody complexes [12]. The cut-off values for urinary DiAcSpm were 243 nmol/g Cre in males and 354 nmol/g Cre in females, respectively.

### Statistical analysis

Differences in participants’ characteristics, laboratory data and tumour markers between the control and cancer participants were examined using the paired *t*-test for continuous variables and the χ^2^ test for dichotomized variables. A *P*-value of <0.05 was considered statistically significant. Cancer staging was based on Union Internationale Contre le Cancer (UICC) criteria. Thereafter, the odds ratios (ORs) and 95% confidence intervals (CIs) for cancer detection were estimated using five logistic regression models. Model 1: NSDT, age and complaints [appetite loss, constipation or diarrhoea, some complaints]; Model 2: NSDT, age and other diseases [hypertension, hyperlipidaemia, cerebral infarction, some other diseases]; Model 3: NSDT, age, Plt, CEA, anti-p53 Ab and DiAcSpm/Cre; Model 4: NSDT, age, hypertension, some other diseases, CEA and some TMs; Model 5: NSDT, age and CEA. The OR for each continuous variable was expressed for one standard deviation (SD) increase. Diagnostic accuracy was calculated as sensitivity, specificity positive predictive value and efficiency of CEA, anti-p53 Ab, DiAcSpm, some TMs, and NSDT of samples, compared with the current diagnosis.

### Worm cultures and strains

*C*. *elegans* strains were cultured at 20°C under standard conditions on NGM plates [13] with *Escherichia coli* NA22, which grows in thick layers that serve as a suitable food source for large-scale worm cultures used for chemotaxis analyses [14–17]. Strains used in this study were wild-type N2 and *odr-3(n2150)*.

### Chemotaxis assays

The chemotaxis assays were conducted using 50–100 approximately synchronized young adults, and the calculation of the chemotaxis index was performed as previously described [16,18]. It was important to maintain room temperature at 23 ± 1°C. Urine samples stored at -20°C were thawed and kept at room temperature just before the assays. Only well-fed animals were used, because starvation affects attraction to cancer urine as well as avoidance of control urine.

### Genetic ablation of sensory neurons

We used mouse caspase-1 (mCasp1) for the ablation of AWC, AWA, ASH and AWB neurons [16,17]. The *ceh-36* [19], *odr-10* [20], *sra-6* [21] and *str-1* [22] promoters were used to drive the expression of mCasp1 in each of these neurons, respectively.

### Calcium imaging

Because urine samples flow in thin tubes in imaging experiments using a microfluidic device, precipitates and solid bodies in the urine had to be removed by centrifugation and filtration (pore size 0.22 μm, MillexGP, Merck Millipore). To monitor the responses of AWC and AWA neurons, YC3.60 [23] was expressed in these neurons by the *odr-1* [24] and *odr-10* [20] promoters, respectively. Calcium imaging was performed as previously described [25–27]. Each animal was immobilized in a microchannel such that the nose of the animal was exposed to a flowing stream containing urine at 10^-1^ dilution. Responses to both control urine and urine from cancer patients were tested in the same individuals. Fluorescent images of YC3.60 were obtained using a Leica DMI3000B microscope equipped with a 40× objective lens and an ORCA-D2 digital camera (Hamamatsu). All images were collected with exposures of 200 ms. Time stacks of AWC or AWA cell bodies were captured and analysed for the emission ratio of YFP to CFP fluorescence using Metamorph software (Molecular devices). The ratio was calculated as YFP intensity/CFP intensity (= R), and the average ratio in a 10-s window (-10–0 s) was set as R0.

## Results and Discussion

**Fig 1:**
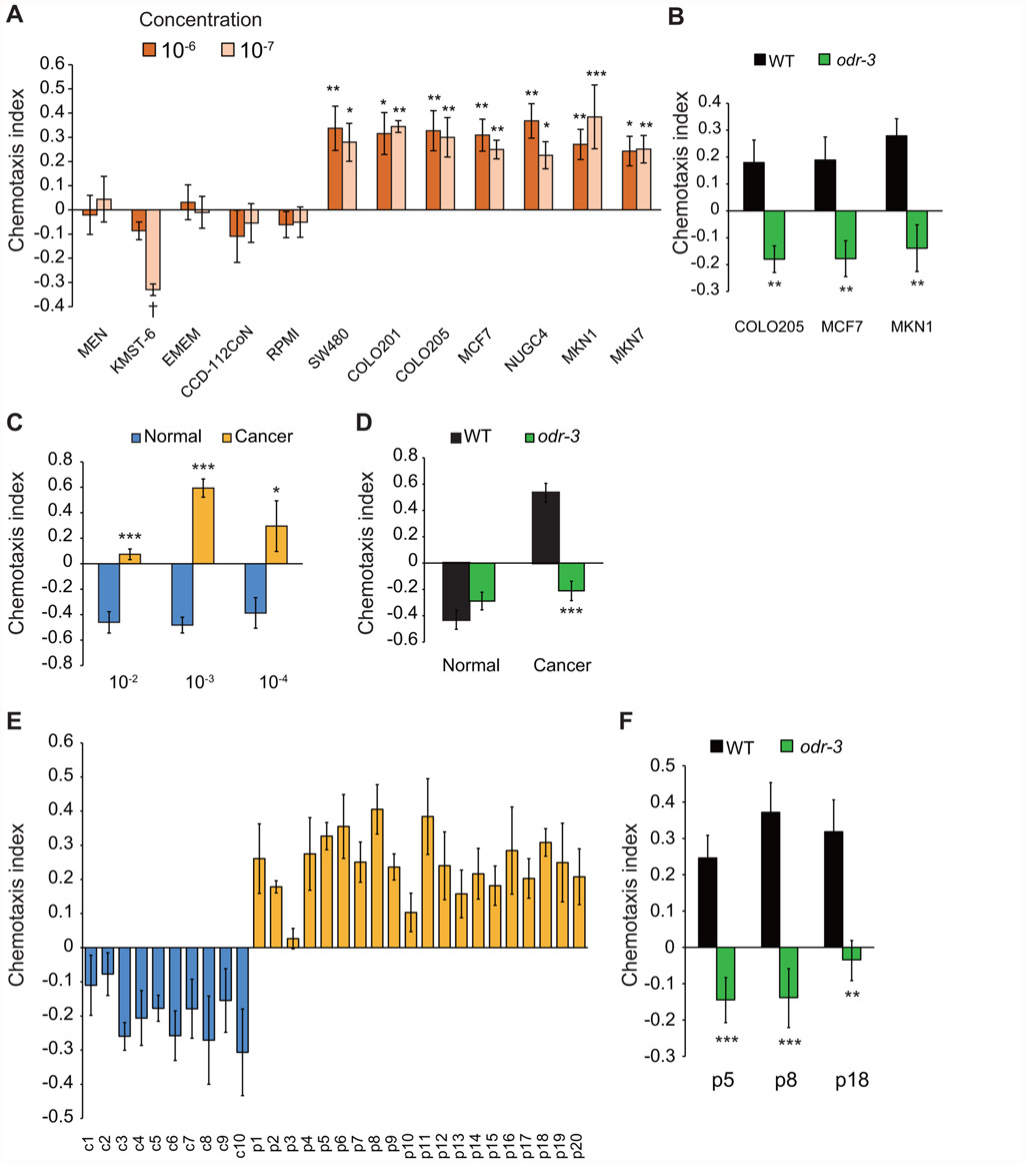
*C*. *elegans* can respond to cancer cell culture medium and cancer tissue, and detect cancer smells in human urine. (A) Chemotaxis of wild-type *C*. *elegans* to 10^-6^ and 10^-7^ dilutions of MEM, EMEM or RPMI medium only, or culture medium from fibroblast (KMST-6 and CCD-112CoN), colorectal cancer (SW480, COLO201 and COLO205), breast cancer (MCF7) or gastric cancer (NUGC4, MKN1 and MKN7) cells (n ≥ 5 assays). (B) Chemotaxis of wild type and *odr-3* mutants (n ≥ 5 assays) in response to a 10^-6^ dilution of conditioned culture medium from colorectal, breast or gastric cancer cells. (C) Chemotaxis of wild type to 10^-2^, 10^-3^ and 10^-4^ dilutions of saline with normal and cancer tissue (n ≥ 5 assays). (D) Chemotaxis to normal and cancer tissue by wild-type and *odr-3* mutants (n ≥ 5 assays). (E) Chemotaxis of wild type to human urine samples from control subjects (blue bars; c1–c10) or cancer patients (orange bars; p1–p20) at 10^-1^ dilution (n = 5 assays). (F) Chemotaxis to urine from cancer patients by wild-type and *odr-3* mutants at 10^-1^ dilution (n ≥ 6 assays). Error bars represent SEM. Significant differences from control samples are indicated by * (*P* < 0.05); ** (*P* < 0.01); *** (*P* < 0.001) by Dunnett’s tests (A) or Student’s *t*-tests (B, C, D, F). † indicates a significant difference (*P* < 0.05) by Student’s *t*-tests (A).

*C*. *elegans* is attracted to or avoids various volatile odorants [18]. To investigate whether *C*. *elegans* detected odours secreted from cancer tissue, we first analysed the response of individual *C*. *elegans* to conditioned medium from cultures of human cancer and fibroblast cell lines. Cell lines derived from human tumours were as follows: colorectal cancer, SW480, COLO201 and COLO205; breast cancer, MCF7; and gastric cancer, NUGC4, MKN1 and MKN7. Cell lines derived from normal human tissues were as follows: embryo fibroblasts, KMST-6 (immortalized using ^60^Co irradiation) and colon fibroblasts, CCD-112CoN. The clear layers at the top of the culture medium were obtained and spotted onto assay plates to assess the chemotactic behaviour of wild-type *C*. *elegans*. To exclude any effect of the smell of the medium, diluted fresh medium was spotted opposite side to the conditioned medium on the assay plates. We found that wild-type *C*. *elegans* showed significant attraction to 10^-6^ or 10^-7^ dilutions of the medium in which cancer cells had been maintained (Fig. 1A). Dose-dependent responses were observed, and peak attraction was induced by the 10^-6^ and 10^-7^ dilutions of the medium (S1 Fig. A). In contrast, wild-type worms were not attracted to a wide range of concentrations of cultured media from human fibroblast cell lines (Fig. 1A and S1B Fig.). Wild-type *C*. *elegans* also showed attraction to media from another cultivation line of cancer cells, but not to those of fibroblasts (S2 Fig.). At higher concentrations, the animals tended to show avoidance behaviour (S1 Fig. A), consistent with our previous report that *C*. *elegans* avoids higher concentrations of attractive odorants [16–18,21,28,29].

In *C*. *elegans*, attractive odorants are sensed by AWC and AWA olfactory neurons [18]. In these sensory neurons, ODR-3 (G protein α) functions as a key component of an olfactory signalling pathway, and *odr-3* mutants cannot respond to attractive odorants [30]. However, ODR-3 is not involved in the responses to other types of attractive chemicals including water-soluble substances. We observed that *odr-3* mutants showed severe defects in attraction to medium from cancer cell lines (Fig. 1B), indicating that *C*. *elegans* senses odorous materials from cultured cancer cells.

Next, we tested whether *C*. *elegans* shows attraction toward human cancer tissue from a cancer patient. We found wild-type *C*. *elegans* exhibited obvious attraction to cancer tissue of 0.1–0.8 mm in diameter from a cancer patient (sigmoid colon cancer, gastric cancer or rectal cancer), but weakly avoided normal tissue from the same participant (S3 Fig.). When cancer or normal tissue was placed at the opposite points each other on the same plate, wild-type *C*. *elegans* preferred cancer tissue (S1 Fig. A).

To quantitatively evaluate this behaviour, we excised cancer and normal tissue of 0.5 cm each from the identical stage II sigmoid colon cancer patient and maintained them in 10 ml saline. Wild-type *C*. *elegans* showed attraction to 10^-3^ or 10^-4^ dilutions of cancer tissue-maintained saline, whereas it showed avoidance to saline with normal tissue (Fig. 1C). Similar to the above result of chemotaxis to cancer cells, the animals tended to show weak attraction at higher concentrations [16]. These results indicate that *C*. *elegans* show attraction to cancer tissue. Moreover, *odr-3* mutants did not exhibit attraction to cancer tissue (Fig. 1D), indicating that *C*. *elegans* senses odorous materials from cancer tissue. To the contrary, the mutants had normal avoidance of normal tissue, which is probably consistent with the previous result ODR-3 mainly mediates attraction whereas partially regulate avoidance and other G proteins are involved in sensing repellents [16,30].

To determine whether the NSDT could be an effective tool for the screening of human cancers, we compared the responses of *C*. *elegans* to the serum and urine of control subjects and patients with cancer (limited characterization). We tested 30 serum and urine samples; 10 samples from controls with no history of cancer and 20 samples from patients with colorectal, gastric or pancreatic cancer (S1 Table). In the response to serum, no difference was observed between samples from control subjects and cancer patients, even though the concentrations of serum were changed (S4 Fig.). This may be due to the presence of other strong odorants or molecules that mask the smell of molecules secreted into the circulation. However, *C*. *elegans* was attracted to the 10^-1^ dilution of urine samples from cancer patients, whereas they avoided all control urine samples (Fig. 1E and S5 Fig.). We tested various concentrations of urine and found that attraction to cancer urine and avoidance of control urine peaked at a 10^-1^ dilution of each (S6 Fig.). This result reveals the possibility that *C*. *elegans* discriminated between urine from controls and cancer patients. Among the patients tested, there were six cases of early-stage cancer (stage 1) (p1, p3, p6, p8, p11 and p12) (S1 Table), suggesting the possibility that the NSDT can be used for screening of early cancer. Moreover, *odr-3* mutants exhibited significant defects in their attraction to urine from cancer patients, further suggesting that *C*. *elegans* senses odours in urine (Fig. 1F).

**Fig 2:**
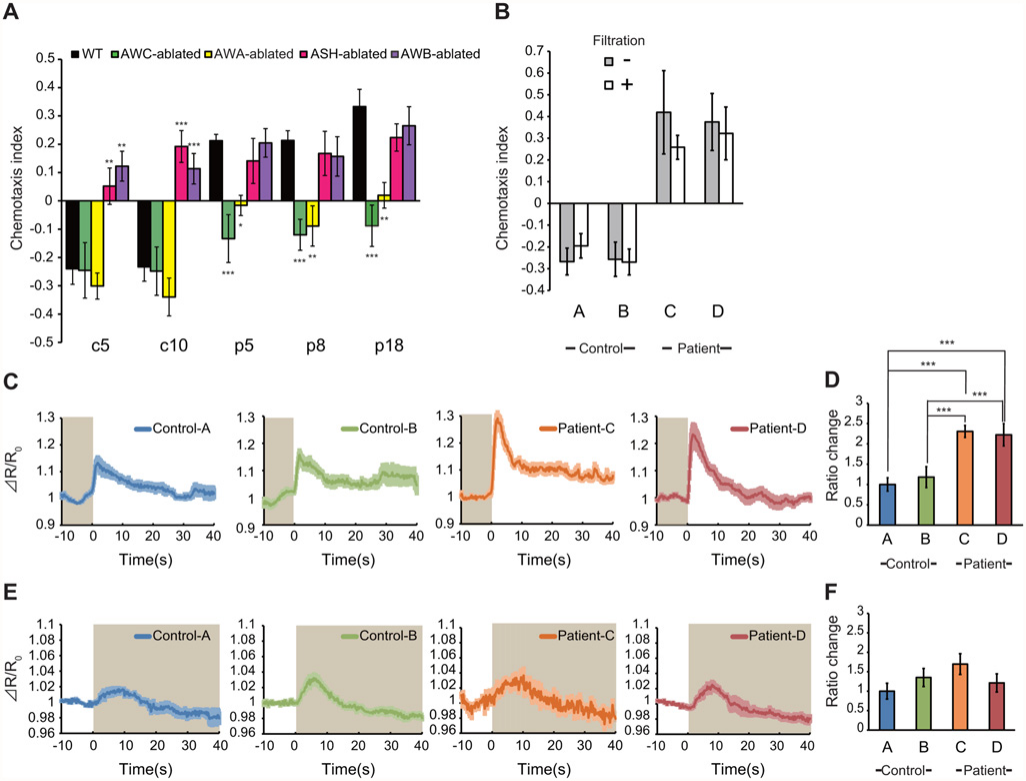
Olfactory neurons of *C*. *elegans* respond to urine from cancer patients. (A) Chemotaxis to urine from controls (c5 and c10) or cancer patients (p5, p8 and p18) in wild-type animals with AWC, AWA, ASH or AWB neuron ablation (n ≥ 5 assays). (B) Chemotaxis of wild-type *C*. *elegans* to urine samples from controls (A and B) or cancer patients (C and D) with or without filtration that were used in imaging experiments (n ≥ 5 assays). Filtration of urine had no significant effect. (C) Calcium responses of AWC olfactory neurons on removal of control or cancer patient urine. (D) Average fluorescence changes in AWC for 10 s following urine removal (n ≥ 8 animals). Values are normalized to the average change in ratio of Control-A. (E) Calcium responses of AWA olfactory neurons after addition of urine from controls or cancer patients. (F) Average fluorescence changes in AWA for 10 s after urine stimulation (n ≥ 8 animals). Values are normalized to the average change in ratio of Control-A. The brown shading indicates that urine was present. Error bars and shaded regions around the curves represent SEM. Significant differences from control samples are indicated by * (*P* < 0.05); ** (*P* < 0.01); *** (*P* < 0.001) as calculated by Dunnett’s tests (A, D, F).

As mentioned above, attractive odorants are sensed by AWC and AWA olfactory neurons in *C*. *elegans*, whereas repellents are sensed by ASH, AWB and other sensory neurons [21,22,31,32]. To determine which sensory neurons sense cancer smells in urine, we analysed the responses of animals with sensory neuron ablation. A previous report indicated that expression of mouse caspase 1 (mCasp1) efficiently kills AWC, AWA, ASH and AWB neurons [16,17]. We found ASH- or AWB- ablation caused defects in avoidance of control urine (Fig. 2A), suggesting that these neurons regulate this behaviour. In contrast, AWC- or AWA-ablated animals showed significant defects in their attraction to urine from cancer patients (Fig. 2A), indicating that the AWC and AWA olfactory neurons mediate attraction to cancer smells in urine.

Based on the results described above, to directly monitor the responses of *C*. *elegans* olfactory neurons to urine from cancer patients, we performed calcium imaging experiments using the genetically encoded calcium indicator, yellow cameleon (YC) 3.60 [23,25–27]. For imaging experiments, we obtained large amounts of urine from controls (A and B) and patients with gastric cancer (C and D), and observed avoidance of or attraction to these urine samples by *C*. *elegans*, respectively (Fig. 2B). For the imaging experiments, precipitates and solid bodies in urine were removed by centrifugation and filtration (see Methods). Such treatment did not affect the chemotactic behaviour of *C*. *elegans* towards urine samples (Fig. 2B). Because our previous results demonstrated that AWC and AWA olfactory neurons mediate *C*. *elegans* attraction to urine from cancer patients (Fig. 2A), we monitored the responses of these neurons following urine stimulation. Previous reports revealed increases in calcium concentration occur in the AWC neurons upon odour removal (an odour-OFF response) [25], whereas that in the AWA neurons is detected after odour addition (an odour-ON response) [33]. We found the AWC olfactory neurons clearly responded to urine from patients with gastric cancer (Fig. 2C and S1 Movie). Increased intracellular Ca^2+^ levels in AWC neurons were observed after removal of urine, while AWC neuronal responses to cancer patient urine were significantly stronger than those to control urine (Fig. 2D), indicating that these neurons play important roles in discriminating between urine from controls and cancer patients. We observed significantly stronger responses of AWC neurons to urine from patients with other types of cancer (rectal and sigmoid colon cancer) (S7 Fig.). AWC neurons also weakly responded to control urine, suggesting the presence of impurities in urine that may slightly activate the AWC neurons. Also, in the AWA olfactory neurons, weak but significant responses to the addition of urine from cancer patients were observed (Fig. 2E and F), although no differences between urine from the controls and the patients were detected in the imaging experiments because the responses of AWA neurons were very faint compared to AWC neurons.

**Fig 3:**
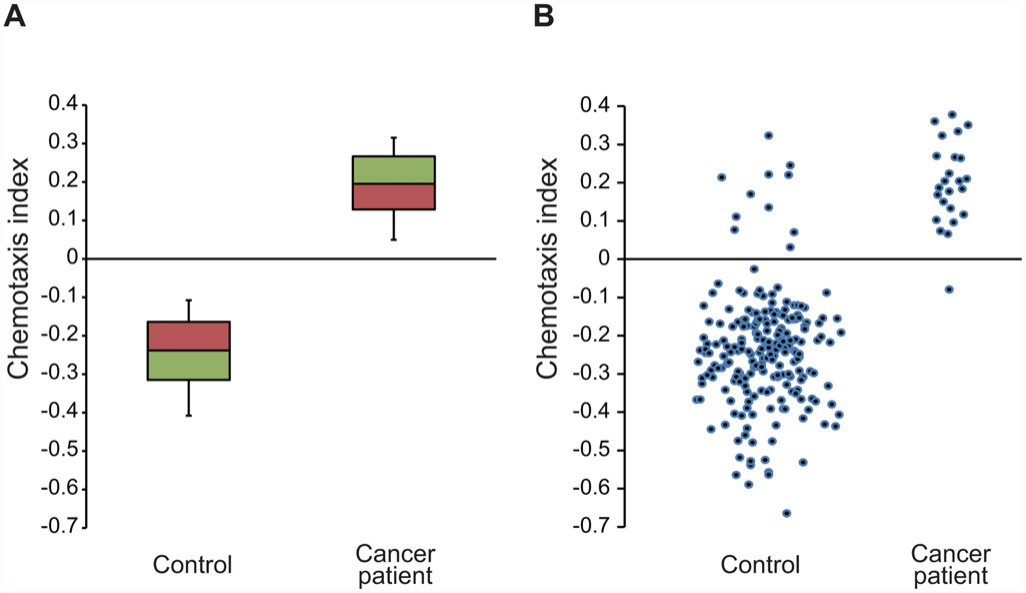
NSDT of 242 urine samples. Box plots (A) and dot plots (B) of chemotactic responses of wild-type *C*. *elegans* to urine samples from control subjects (n = 218) or cancer patients (n = 24). Whiskers indicate 10th and 90th percentiles.

To examine the accuracy of the NSDT, we analysed 242 urine samples; 218 control samples and 24 samples from cancer patients (extended characterization) (S2 Table). All the urine samples were diluted to 10^-1^ concentration and chemotaxis assays were performed three times for each sample. When the result of three times assays straddled 0, three times assays were performed again for the same sample. *C*. *elegans* showed attraction to various cancer patient urine samples (23/24) (Fig. 3). The sensitivity of the NSDT was 95.8% (Table 1). However, *C*. *elegans* exhibited avoidance of most control urine samples (207/218) (Fig. 3). The specificity of the NSDT was 95.0%, and the positive predictive value and efficiency of the test were 67.6% and 95.0%, respectively (Table 1). We also analysed the patient questionnaire and other existing tumour markers including serum CEA [10], serum anti-p53 antibody (anti-p53 Ab) [11] and urine N^1^, N^12^-diacetylspermine (DiAcSpm) [12] in the same participants. Compared with these existing tumour markers, the specificity of the NSDT was much higher (Table 1). Nevertheless, the sensitivity of the existing tumour markers tended to be lower with earlier-stage cancer, yet that of the NSDT remained high for all stages of cancer. We also measured urine creatinine concentration, which was used as an indicator of urine concentration for all samples. However, the responses of *C*. *elegans* to urine were not associated with urine concentration, suggesting that cancer odour secretion is independent. Although DiAcSpm levels were obviously increased in the urine of all three pregnant women tested, the NSDT was not affected by pregnancy. Also, chemotaxis to urine had no correlation with the sex of the participants, most physical complaints and diseases other than cancer including diabetes, or the type of medicine administered (S2 Table). There were significant differences in the NSDT, participant age, appetite loss, constipation or diarrhoea, some complaints, hypertension, hyperlipidaemia, cerebral infarction, some other diseases, blood platelets, CEA, and some tumour markers (TMs) between cancer patients and control participants (S2 Table). Logistic regression analyses were performed to identify independent factors for cancer detection and the effect of NSDT was examined via these analyses. Appetite loss, constipation or diarrhoea, some complaints, hypertension, hyperlipidaemia, cerebral infarction, some other diseases, blood platelets, anti-p53 Ab and DiAcSpm/Cre were not significantly associated with cancer detection (S3 Table). As a result, the NSDT and participant age were independent variables for cancer detection in all models in the logistic analyses. The odds ratio for 0.2 increase in the NSDT was between approximately 30.4 to 49.8 for all models (S3 Table). These results indicate that the NSDT can specifically detect cancer. The participants in this test included patients with early cancer (stage 0 or 1) (Table 1), indicating that the NSDT can detect early-stage cancer. Note that in five participants that were not categorized as cancer patients when urine was obtained in 2011, the NSDT identified the urine of these participants as cancer-positive; cancer was found during the subsequent two years. The NSDT could detect various types of cancer tested in this study including oesophageal, gastric, colorectal, breast, pancreatic and prostate cancer at all stages (Fig. 3 and Table 1), suggesting that this test can be used for inclusive cancer screening.

**Table 1:** Accuracy of tumour markers in extended characterization.

	Stage	n	CEA	Anti-p53 Ab	DiAcSpm	Some TMs	NSDT
Oesophageal ca.	0	1	0	0	0	0	1
	Total	1	0	0	0	0	1
Gastric ca.	I	4	0	1	0	1	4
	IV	1	1	0	0	1	1
	Total	5	1	1	0	2	5
Colorectal ca.	0	2	1	0	0	1	2
	I	1	0	0	0	0	1
	II	2	0	1	0	1	2
	III	4	1	0	1	2	4
	IV	1	1	1	1	1	1
	Total	10	3	2	2	5	10
Breast ca.	I	2	0	1	0	1	2
	II	3	1	0	0	1	3
	Total	5	1	1	0	2	5
Pancreatic ca.	IV	1	1	0	1	1	1
	Total	1	1	0	1	1	1
Bile duct ca.	I	1	0	0	1	1	0
	Total	1	0	0	1	1	0
Prostate ca.	I	1	0	0	0	0	1
	Total	1	0	0	0	0	1
All cancers	0	3	1	0	0	1	3
	I	9	0	2	1	3	8
	II	5	1	1	0	2	5
	III	4	1	0	1	2	4
	IV	3	3	1	2	3	3
	Total	24	6	4	4	11	23

Sensitivity	Stage	CEA	Anti-p53 Ab	DiAcSpm	Some TMs	NSDT
	0	33.3%	0.0%	0.0%	33.3%	**100.0%**
	I	0.0%	22.2%	11.1%	33.3%	**88.9%**
	II	20.0%	20.0%	0.0%	40.0%	**100.0%**
	III	25.0%	0.0%	25.0%	50.0%	**100.0%**
	IV	100%	33.3%	66.7%	100%	**100.0%**
	Total	25.0%	16.7%	16.7%	45.8%	**95.8%**
Specificity		96.3%	86.2%	95.4%	83.9%	**95.0%**
Positive predictive value		42.9%	17.4%	28.6%	23.9%	**67.6%**
Efficiency		89.3%	83.9%	87.6%	80.2%	**95.0%**

The type and stage of cancer from which serum CEA, serum anti-p53 antibody, urinary DiAcSpm/Cre, some TMs and NSDT were measured or detected in 24 cancer patients are shown. Some TMs indicate that positive results were found for at least one tumour marker: CEA, Anti-p53 Ab or DiAcSpm. Sensitivity, specificity, positive predictive value and efficiency are also demonstrated. Such values of the NSDT were markedly higher than those of other existing tumour markers.

In the present study, we show that *C*. *elegans* discriminated efficiently between the urine of cancer patients and control subjects. Nevertheless, the identification of unique cancer odours in urine and the confirmation of the responses of *C*. *elegans* is required to support the conclusion that *C*. *elegans* detects cancer smells in urine. We believe that the data presented here indicate that using *C*. *elegans* shows promise for cancer diagnosis. Experienced clinicians know that the human nose is a valuable tool in bedside diagnosis, but the ability of humans to diagnose disease by smell has only very rarely been the subject of quantitative studies. Recently, many scent detection studies have been performed with animals [2–6,34], gas chromatography/ mass spectroscopy (GC/MS) [6,35–39] or electronic noses (Enoses) [40–42]. Cancer detection by GC/MS or Enoses is problematic with regard to detection sensitivity, the noise of the volatile organic compounds that exist in the environment, and high cost. Although no direct comparison studies have been performed, dogs appear to outperform GC/MS or Enoses [43]. However, the intelligence and concentration of these animals is disadvantageous in cancer screening that requires the mechanical inspection of numerous samples. Therefore, we postulated that if we could separate scent detection from intelligence we could investigate the relationship between diseases and scents. We observed the keen scent detection of the nematode *C*. *elegans*, which is not influenced by or reliant upon intelligence.

To detect cancer smells more precisely and quantitatively, it is necessary to identify specific cancer odours and their receptors. Several volatile organic compounds have been identified as candidate substances for cancer detection using gas chromatography/mass spectroscopy (GC/MS) [6,35–39] or Enoses [40–42] in cancer cell lines, urine or exhaled breath. But while these data are promising, they are preliminary findings. By using *C*. *elegans* to test large numbers of samples, comprehensive screening of cancer odours can be performed efficiently. Moreover, olfactory receptors that bind cancer smells have not yet been identified, because molecular and genetic analyses are difficult or unavailable in higher organisms such as dogs. Given the power of genetics and molecular techniques in *C*. *elegans*, our results provide a platform of efficient identification of receptors related to cancer odours. We have already begun attempts to identify cancer odours and their receptors using the NSDT. Identification of these may lead not only to the elucidation of metabolic mechanisms of cancer cells, thus contributing to the study of anticancer agents, but also to the development of a convenient kit to detect cancer using exhaled breath or urine and a novel anti-cancer drug delivery system with no side-effects, both based on the binding between the cancer odour and its receptor.

*C*. *elegans* showed attraction to the urine samples regardless of cancer type. However, the NSDT cannot identify the organs harbouring cancer cells. Therefore, combination of the NSDT with existing methods of cancer diagnosis and new methods such as metabolomic analyses will enable complementary sensitivity and specificity. The NSDT has outstanding characteristics such as high accuracy, low cost, painlessness, convenience and speed, and use of urine without restriction of meals and activities. Notably, the special features of the NSDT are its high cost-performance and low set-up costs. It will be necessary to confirm judgement criteria for practical use and to improve sensibility of worms to cancer smells for application of NSDT to diagnosis.

## Supporting Information

S1 Fig Chemotaxis of wild-type C. elegans in response to media from cultures of human cancer and fibroblast cell lines. Chemotactic response of wild-type *C*. *elegans* to various concentrations (10^-0^–10^-9^) of cultured media from the human cancer cell lines COLO205 and MKN1 (A) and the human fibroblast cell lines KMST-6 and CCD-112CoN (B), n ≥5 assays. Error bars represent the SEM. Significant differences from controls are indicated by * (*P* < 0.05), ** (*P* < 0.01). Dunnett (A) or Student *t* (B) tests.
(PDF)

S2 Fig Chemotaxis of wild-type *C*. *elegans* to media from another cultivation line of cancer and fibroblast cells. Chemotaxis of wild-type *C*. *elegans* to 10^-6^ and 10^-7^ dilutions of MEM, EMEM or RPMI medium only, or the medium from another cultivation line of fibroblast (KMST-6 and CCD-112CoN), colorectal cancer (SW480, COLO201 and COLO205), breast cancer (MCF7) or gastric cancer (NUGC4, MKN1 and MKN7) cells (n ≥ 5 assays). Error bars represent SEM. Significant differences from control samples are indicated by * (*P* < 0.05); ** (*P* < 0.01); *** (*P* < 0.001) by Dunnett’s tests or † (*P* < 0.05) by Student’s t-tests.
(PDF)

S3 Fig Chemotaxis of wild-type *C*. *elegans* in response to cancer tissues. Representative images of chemotaxis of *C*. *elegans* to cancer or normal tissues (A: sigmoid colon cancer, B: gastric cancer, C: rectal cancer). Cancer or normal tissue 0.1–0.8 mm in diameter was placed at the point indicated in the figures. Sodium azide (0.5 μl, 1 M) was spotted at ‘+’ and opposite points. One hour after the worms were placed at the start points (arrowheads), the plates were photographed. A red rectangle indicates an enlarged view (A). The bar graph shows the average chemotaxis indices toward sigmoid colon cancer tissue, normal tissue and the assays of preference for cancer or normal tissue (n = 3 assays). Error bars represent the SEM. An asterisk indicates a significant difference (*P* < 0.05, Student’s *t* test).
(PDF)

S4 Fig Chemotaxis of *C*. *elegans* in response to serum samples from control participants and patients with cancer. Chemotaxis of wild-type *C*. *elegans* in responses to various dilutions (10^-0^, 10^-1^, 10^-3^ and 10^-5^) of serum samples from control participants (c1, c2 and c3) and patients with cancer (p2, p5, p8, p17 and p18), n ≥ 5 assays. Characteristics of participants are shown in S1 Table.
(PDF)

S5 Fig Chemotaxis of wild-type *C*. *elegans* in response to urine from a control participant and a cancer patient. Representative images of the chemotactic responses of wild-type *C*. *elegans* to 1 μl of a 10^-1^ dilution of urine from a control participant and a cancer patient. Urine was spotted at the ‘+’ points, and 0.5 μl of 1 M sodium azide was spotted at the ‘+’ and opposite points. The plates were photographed 1 h after the worms were placed at the start points (arrowheads).
(PDF)

S6 Fig Chemotaxis of wild-type *C*. *elegans* in response to various concentrations of urine from control participants and cancer patients. Chemotactic responses of wild-type *C*. *elegans* to dilutions (10^-0^, 10^-1^, 10^-2^, 10^-3^ and 10^-5^) of urine samples from control participants (c1, c2 and c3) and cancer patients (p2, p5, p8, p17 and p18), n ≥ 5 assays. Background characteristics of participants are shown in S1 Table.
(PDF)

S7 Fig Responses of AWC neurons to urine from patients with cancer. (A) Chemotaxis of wild-type *C*. *elegans* in response to urine samples from control (E) or cancer patients (F, rectal cancer and G, sigmoid colon cancer), with or without filtration, which were used in the imaging experiments (n ≥ 5 assays). (B) Average fluorescence changes in AWC neurons for 10 s following urine removal (n ≥ 7 worms). Values are normalized to the average ratio change of Control-E. Error bars represent the SEM. Significant differences from the control are indicated by *** (*P* < 0.001), * (*P* < 0.05) as calculated by Student’s *t*-tests.
(PDF)

S1 Movie AWC neurons strongly respond to urine from cancer patients. Changes in concentration of intracellular Ca^2+^ in AWC olfactory neurons after removal of cancer patient urine (Patient C in Fig. 2C) are shown. At time 0, urine was removed. Images are color-coded with green to indicate low fluorescence ratios and red to indicate high fluorescence ratios and an increase in intracellular Ca^2+^ concentration.
(MOV)

S1 Table Limited background characteristics of participants. Sex, age, cancerous organ, cancer stage and CEA value for participants (10 controls and 20 cancer patients) are shown. M or F indicates male or female, respectively. A, D, S, and ca. indicates ascending, descending, sigmoid, and cancer, respectively. CEA indicates carcinoembryonic antigen. Cancer staging was based on Union Internationale Contre le Cancer (UICC) criteria.
(PDF)

S2 Table Extended background characteristics of participants. Data for sex, age, patient cancer history, number of patients with physical complaints and diseases other than cancer, and laboratory data are shown. NS, not significant. *P* values were calculated using the Student *t* test or chi-square test. WBC, Hgb, Plt, CRP, CEA, anti-p53 Ab and urine DiAcSpm/Cre indicate white blood cells, hemoglobins, blood platelets, C-reactive protein, carcinoembryonic antigen, anti-p53 antibody and urine N1, N12-diacetylspermine/creatinine, respectively. Certain complaints or other diseases indicate that the subject declared at least one complaint or disease other than cancer. Some TMs indicate a positive result for at least one of the tumour markers as follows: CEA, Anti-p53 Ab or DiAcSpm.
(PDF)

S3 Table Multivariate adjusted odds ratio for cancer detection. The ORs and 95% confidence intervals (CIs) for cancer detection were estimated using five logistic regression models. Model 1: NSDT, age and complaints (appetite loss, constipation or diarrhoea, some complaints); Model 2: NSDT, age and other diseases (hypertension, hyperlipidaemia, cerebral infarction, some other diseases); Model 3: NSDT, age, Plt, CEA, anti-p53 Ab and DiAcSpm/Cre; Model 4: NSDT, age, hypertension, some other diseases, CEA and some positive TMs; Model 5: NSDT, age and CEA. The OR for each continuous variable was expressed as one standard deviation (SD) increase (0.20 for NSDT; 13.4 for age; 5.43 for Plt, 2.18 for CEA, 5.48 for anti-p53 Ab, 618 for DiAcSpm/Cre). Significant differences from control samples are indicated by * (*P* < 0.05), ** (*P* < 0.01), and *** (*P* < 0.001).
(PDF)
