# Supplementary material for: A Highly Accurate Inclusive Cancer Screening Test Using Caenorhabditis elegans Scent Detection
Source: PLoS One. 2015 Mar 11;10(3):e0118699. doi: 10.1371/journal.pone.0118699 (PMC4356513; doi:10.1371/journal.pone.0118699)
Supplement: S1 Fig — Chemotactic response of wild-type C. elegans to various concentrations (10-0–10-9) of cultured media from the human cancer cell lines COLO205 and MKN1 (A) and the human fibroblast cell lines KMST-6 and CCD-112CoN (B), n ≥5 assays. Error bars represent the SEM. Significant differences from controls are indicated by * (P < 0.05), ** (P < 0.01). Dunnett (A) or Student t (B) tests. (PDF) [file pone.0118699.s001.pdf]

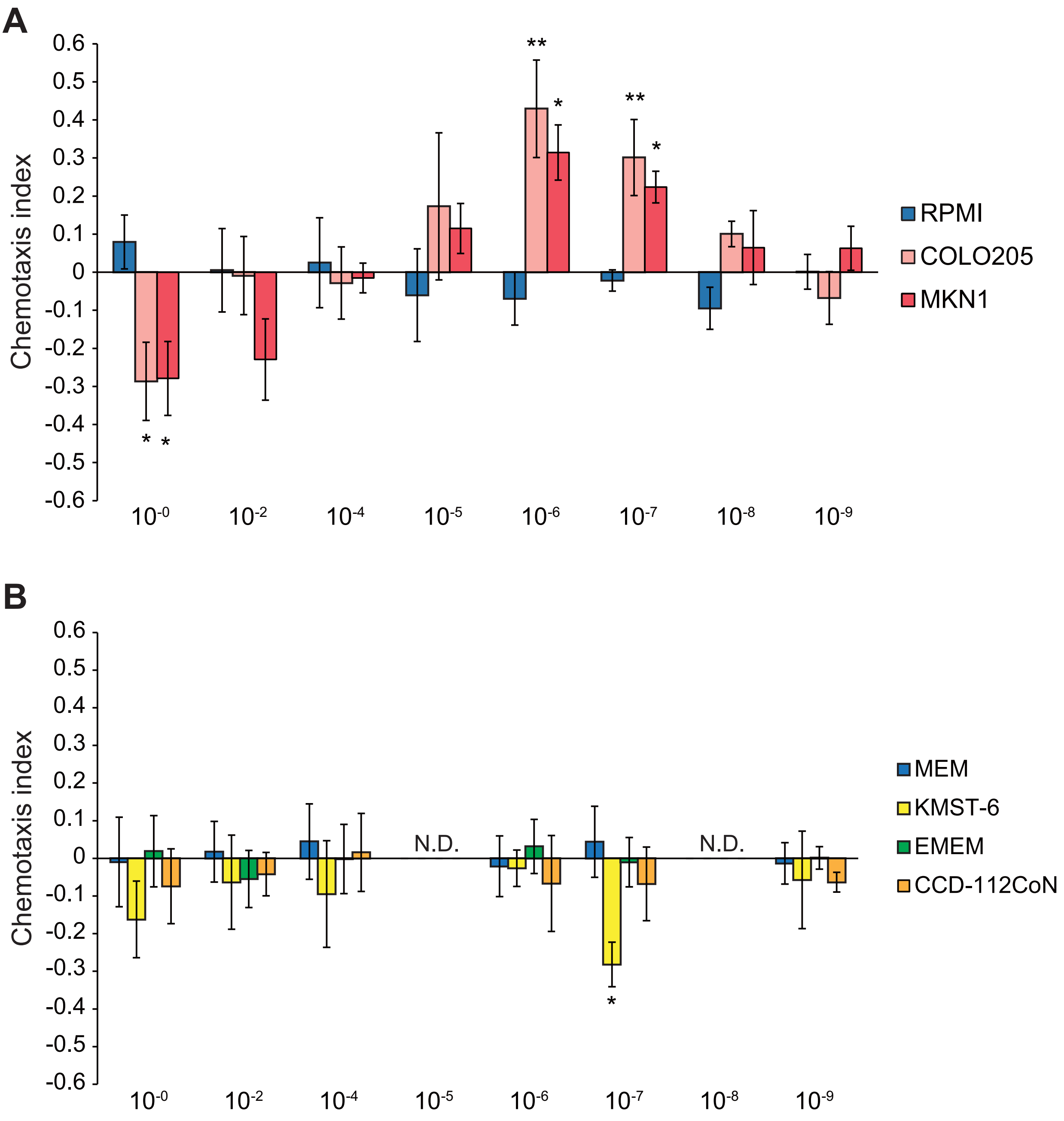

S1 Fig. Chemotaxis of wild-type *C. elegans* in response to media from cultures of human cancer and fibroblast cell lines.

Chemotactic response of wild-type *C. elegans* to various concentrations (10^{-0}–10^{-9}) of cultured media from the human cancer cell lines COLO205 and MKN1 (A) and the human fibroblast cell lines KMST-6 and CCD-112CoN (B), n ≥5 assays. Error bars represent the SEM. Significant differences from controls are indicated by * ($P < 0.05$), ** ($P < 0.01$). Dunnett's (A) or Student's *t* (B) tests.
