# Supplementary material for: A Highly Accurate Inclusive Cancer Screening Test Using Caenorhabditis elegans Scent Detection
Source: PLoS One. 2015 Mar 11;10(3):e0118699. doi: 10.1371/journal.pone.0118699 (PMC4356513; doi:10.1371/journal.pone.0118699)
Supplement: S2 Fig — Chemotaxis of wild-type C. elegans to 10-6 and 10-7 dilutions of MEM, EMEM or RPMI medium only, or the medium from another cultivation line of fibroblast (KMST-6 and CCD-112CoN), colorectal cancer (SW480, COLO201 and COLO205), breast cancer (MCF7) or gastric cancer (NUGC4, MKN1 and MKN7) cells (n ≥ 5 assays). Error bars represent SEM. Significant differences from control samples are indicated by * (P < 0.05); ** (P < 0.01); *** (P < 0.001) by Dunnett’s tests or † (P < 0.05) by Student’s t-tests. (PDF) [file pone.0118699.s002.pdf]

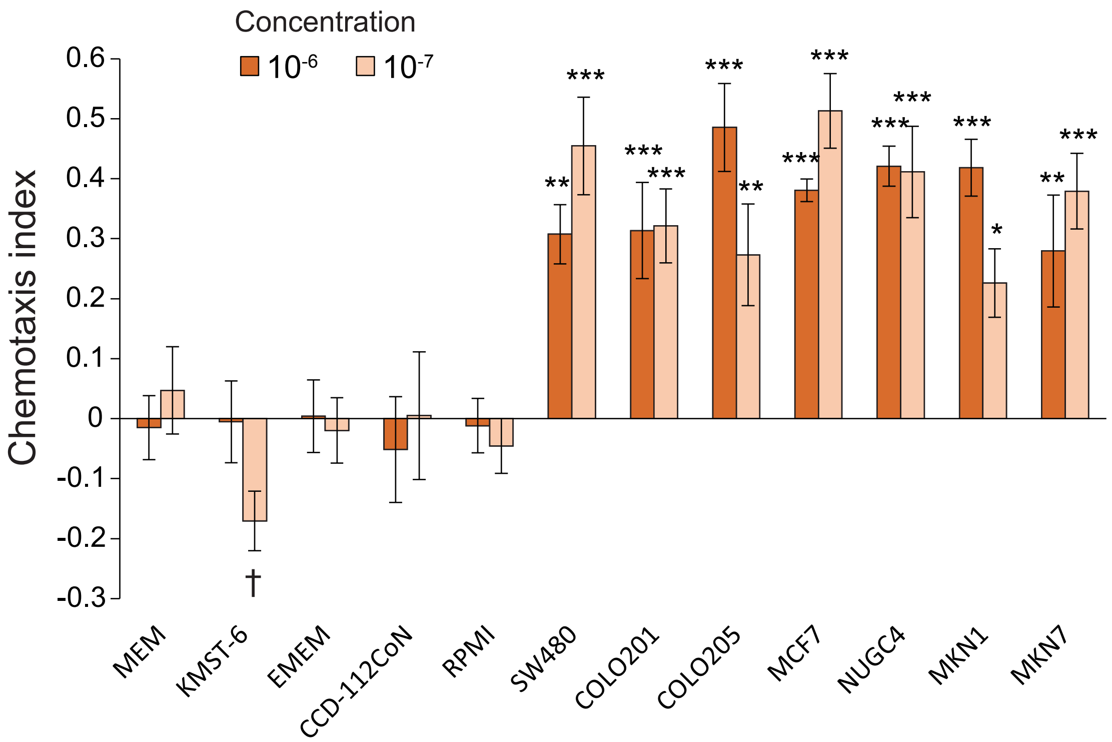

S2 Fig. Chemotaxis of wild-type *C. elegans* to media from another cultivation line of cancer and fibroblast cells.

Chemotaxis of wild-type *C. elegans* to 10^{-6} and 10^{-7} dilutions of MEM, EMEM or RPMI medium only, or the medium from another cultivation line of fibroblast (KMST-6 and CCD-112CoN), colorectal cancer (SW480, COLO201 and COLO205), breast cancer (MCF7) or gastric cancer (NUGC4, MKN1 and MKN7) cells ($n \geq 5$ assays). Error bars represent SEM. Significant differences from control samples are indicated by * ($P < 0.05$); ** ($P < 0.01$); *** ($P < 0.001$) by Dunnett' s tests or † ($P < 0.05$) by Student' s *t*-tests.
