# Supplementary material for: A Highly Accurate Inclusive Cancer Screening Test Using Caenorhabditis elegans Scent Detection
Source: PLoS One. 2015 Mar 11;10(3):e0118699. doi: 10.1371/journal.pone.0118699 (PMC4356513; doi:10.1371/journal.pone.0118699)
Supplement: S3 Fig — Representative images of chemotaxis of C. elegans to cancer or normal tissues (A: sigmoid colon cancer, B: gastric cancer, C: rectal cancer). Cancer or normal tissue 0.1–0.8 mm in diameter was placed at the point indicated in the figures. Sodium azide (0.5 μl, 1 M) was spotted at ‘+’ and opposite points. One hour after the worms were placed at the start points (arrowheads), the plates were photographed. A red rectangle indicates an enlarged view (A). The bar graph shows the average chemotaxis indices toward sigmoid colon cancer tissue, normal tissue and the assays of preference for cancer or normal tissue (n = 3 assays). Error bars represent the SEM. An asterisk indicates a significant difference (P < 0.05, Student’s t test). (PDF) [file pone.0118699.s003.pdf]

A

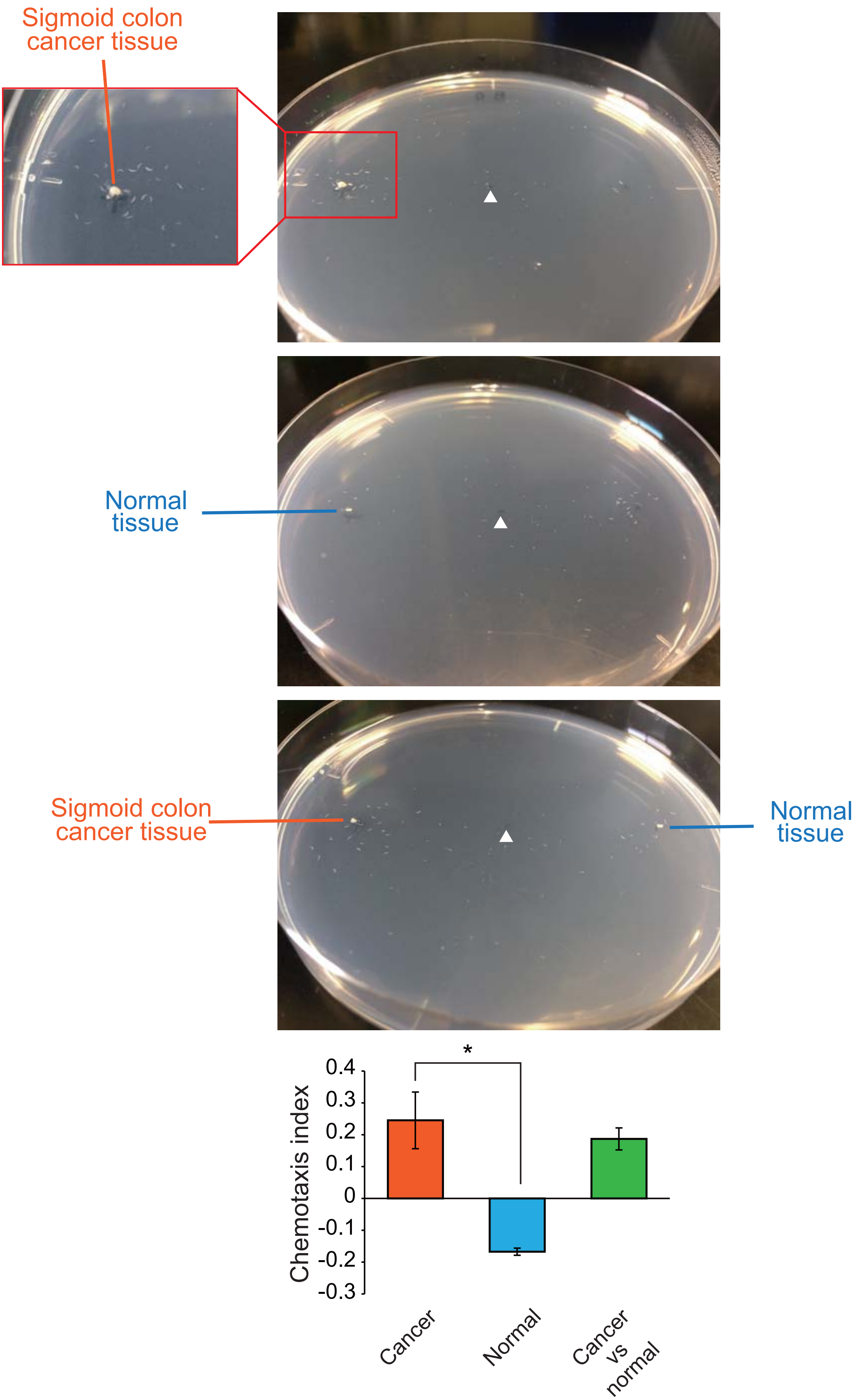

Sigmoid colon cancer tissue
Normal tissue
Sigmoid colon cancer tissue
Normal tissue
*
0.4
0.3
0.2
0.1
0
-0.1
-0.2
-0.3
Chemotaxis index
Cancer
Normal
Cancer vs normal

B

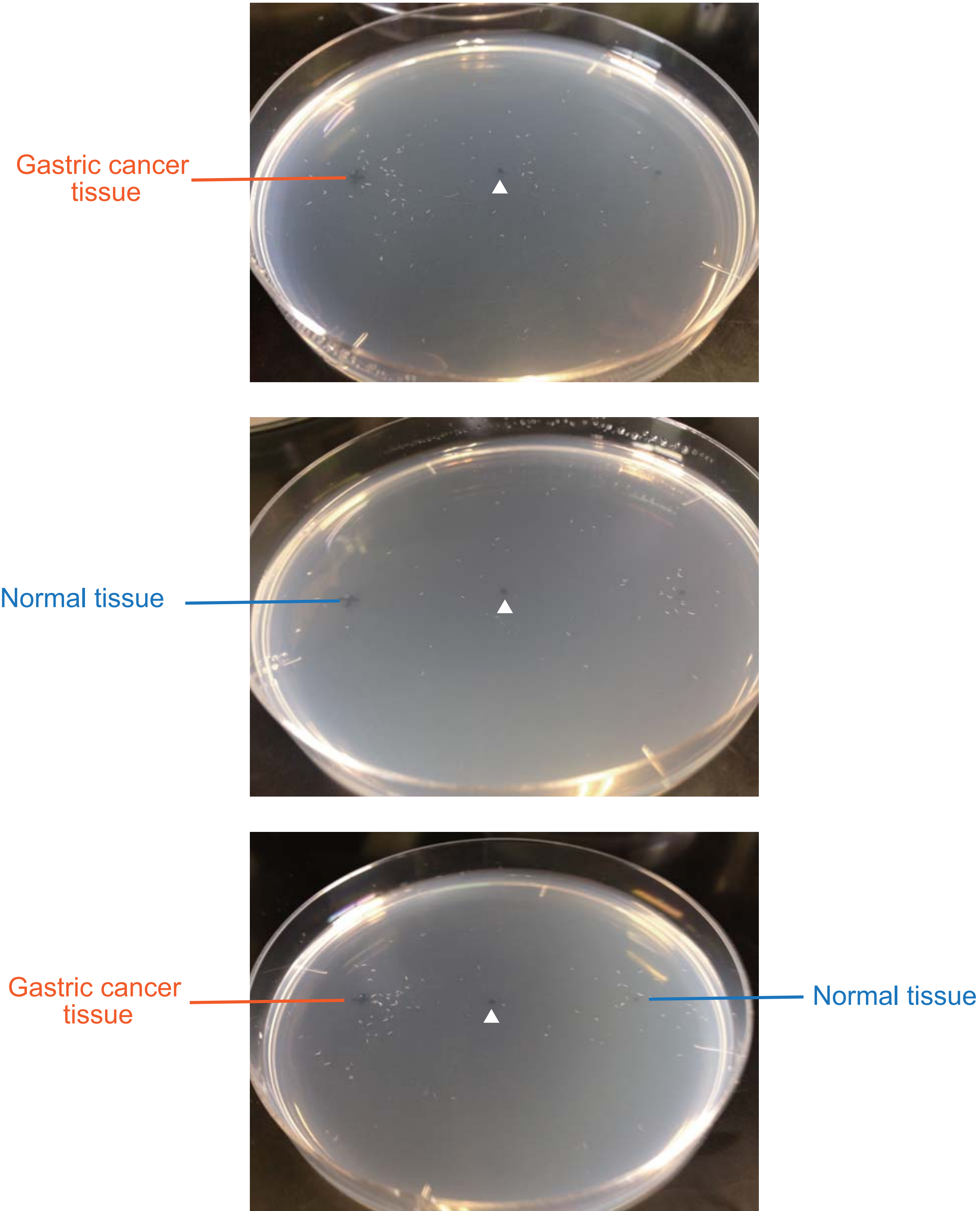
Gastric cancer tissue
Normal tissue
Gastric cancer tissue
Normal tissue

C

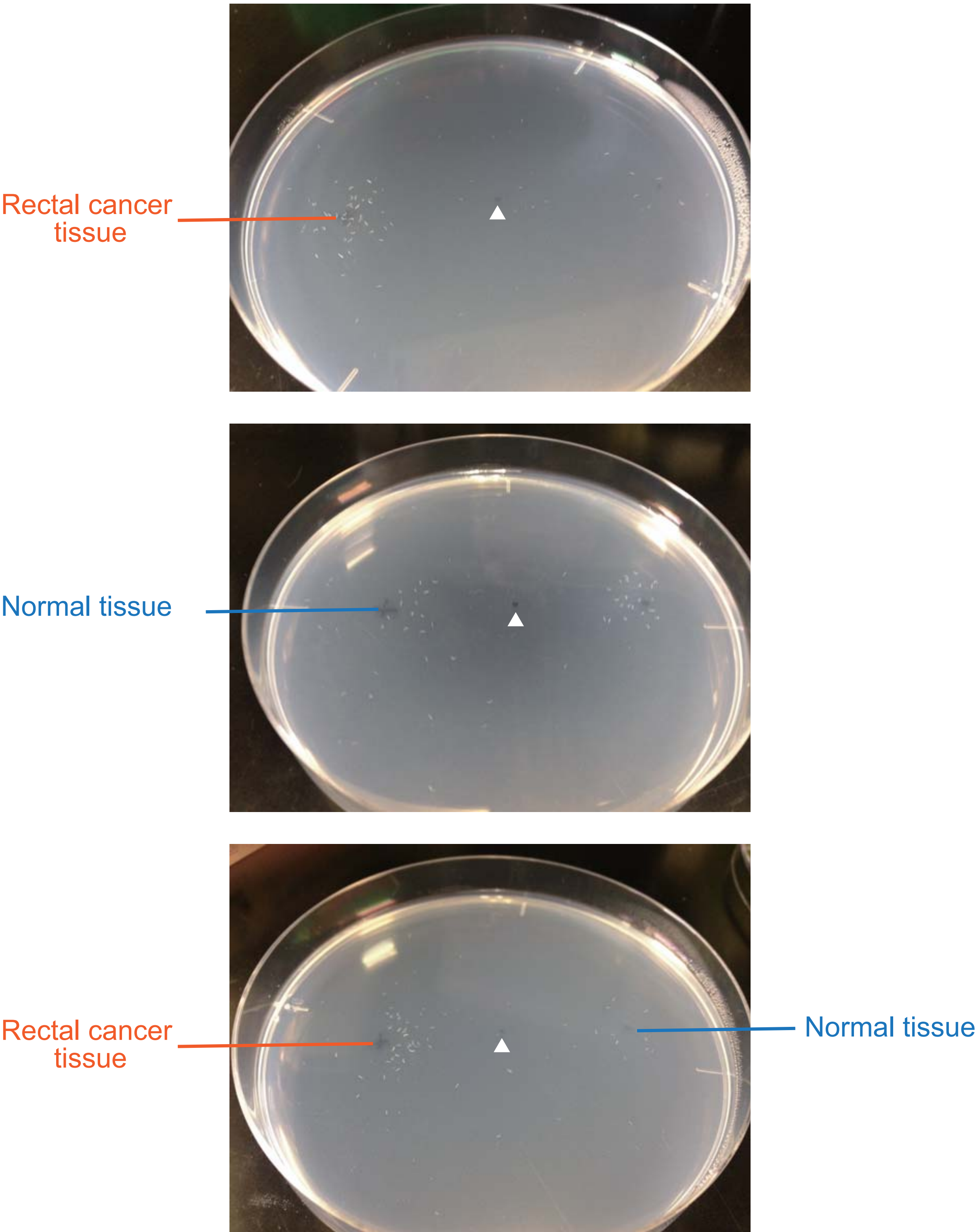
Rectal cancer tissue
Normal tissue
Rectal cancer tissue
Normal tissue

S3 Fig. Chemotaxis of wild-type *C. elegans* in response to cancer tissues.

Representative images of chemotaxis of *C. elegans* to cancer or normal tissues (A: sigmoid colon cancer, B: gastric cancer, C: rectal cancer). Cancer or normal tissue 0.1–0.8 mm in diameter was placed at the point indicated in the figures. Sodium azide (0.5 μl, 1 M) was spotted at '+' and opposite points. One hour after the worms were placed at the start points (arrowheads), the plates were photographed. A red rectangle indicates an enlarged view (A). The bar graph shows the average chemotaxis indices toward sigmoid colon cancer tissue, normal tissue and the assays of preference for cancer or normal tissue (n = 3 assays). Error bars represent the SEM. An asterisk indicates a significant difference ($P < 0.05$, Student' s *t*-test).
