# Supplementary material for: A Highly Accurate Inclusive Cancer Screening Test Using Caenorhabditis elegans Scent Detection
Source: PLoS One. 2015 Mar 11;10(3):e0118699. doi: 10.1371/journal.pone.0118699 (PMC4356513; doi:10.1371/journal.pone.0118699)
Supplement: S4 Fig — Chemotaxis of wild-type C. elegans in responses to various dilutions (10-0, 10-1, 10-3 and 10-5) of serum samples from control participants (c1, c2 and c3) and patients with cancer (p2, p5, p8, p17 and p18), n ≥ 5 assays. Characteristics of participants are shown in S1 Table. (PDF) [file pone.0118699.s004.pdf]

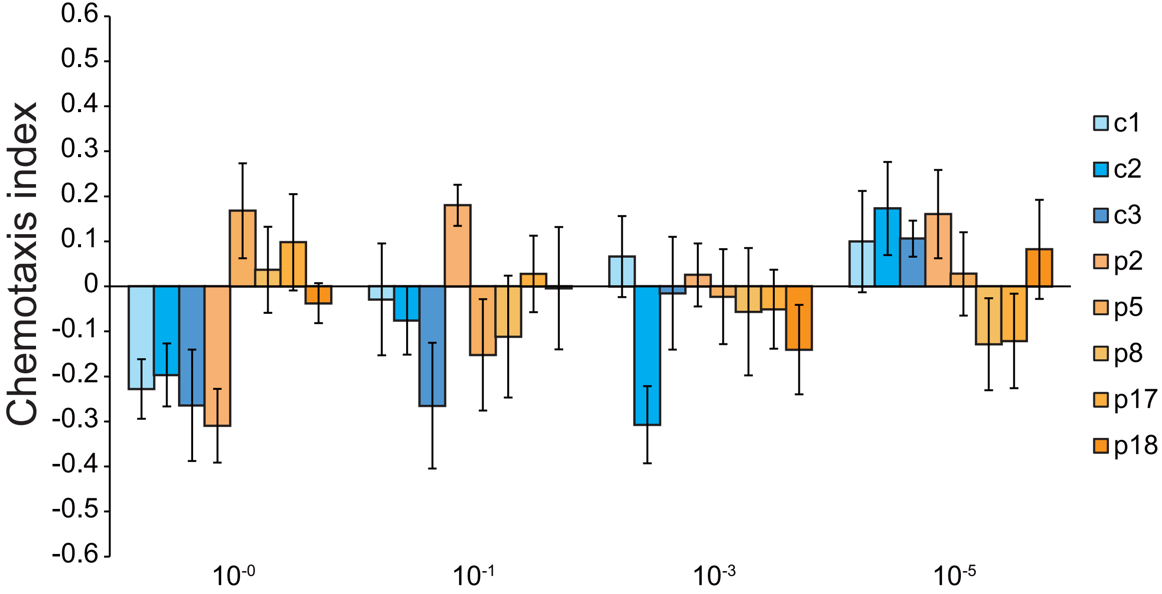

S4 Fig. Chemotaxis of *C. elegans* in response to serum samples from control participants and patients with cancer.

Chemotaxis of wild-type *C. elegans* in responses to various dilutions (10^{-0}, 10^{-1}, 10^{-3} and 10^{-5}) of serum samples from control participants (c1, c2 and c3) and patients with cancer (p2, p5, p8, p17 and p18), n ≥ 5 assays. Characteristics of participants are shown in S1 Table.
