# Supplementary material for: A Highly Accurate Inclusive Cancer Screening Test Using Caenorhabditis elegans Scent Detection
Source: PLoS One. 2015 Mar 11;10(3):e0118699. doi: 10.1371/journal.pone.0118699 (PMC4356513; doi:10.1371/journal.pone.0118699)
Supplement: S5 Fig — Representative images of the chemotactic responses of wild-type C. elegans to 1 μl of a 10-1 dilution of urine from a control participant and a cancer patient. Urine was spotted at the ‘+’ points, and 0.5 μl of 1 M sodium azide was spotted at the ‘+’ and opposite points. The plates were photographed 1 h after the worms were placed at the start points (arrowheads). (PDF) [file pone.0118699.s005.pdf]

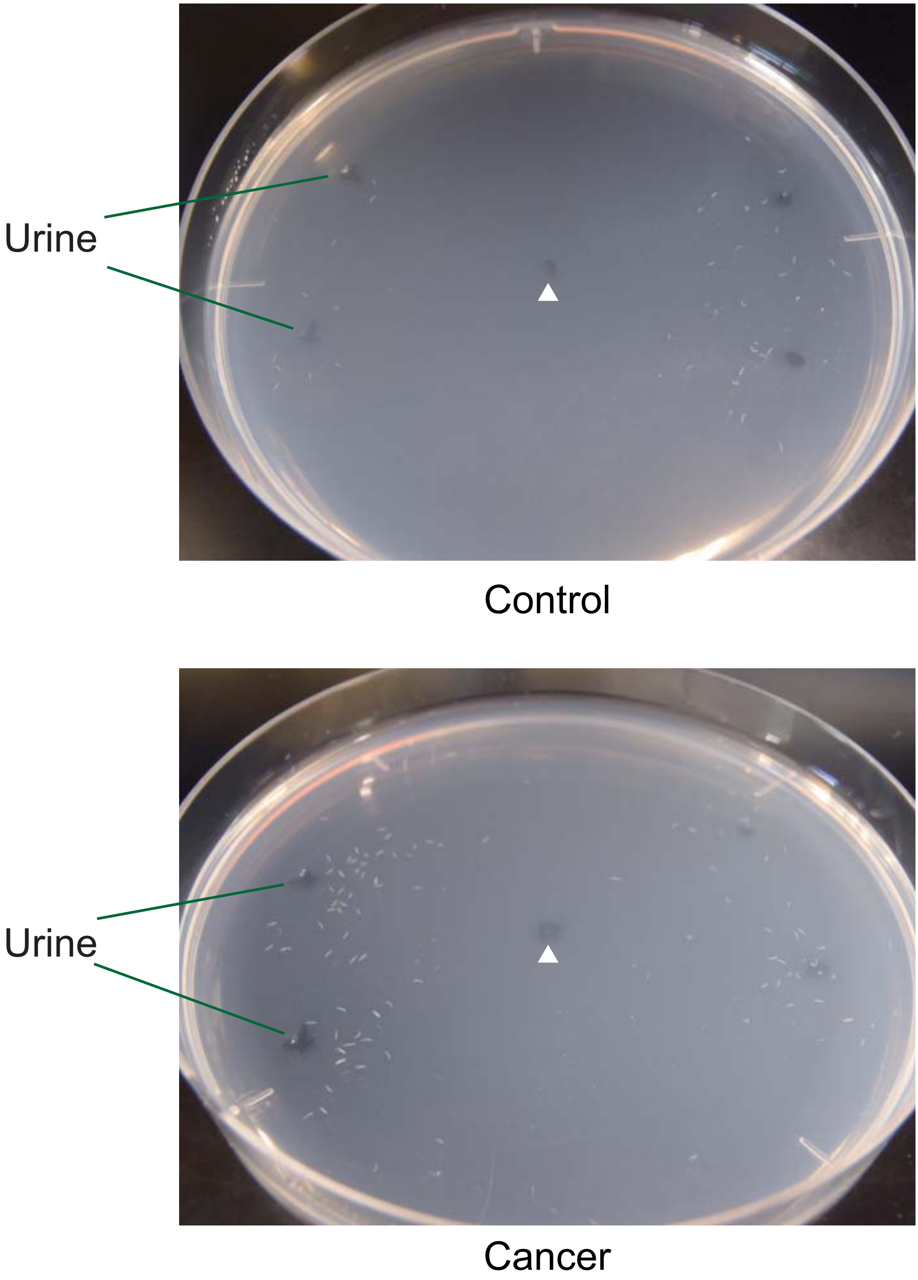

S5 Fig. Chemotaxis of wild-type *C. elegans* in response to urine from a control participant and a cancer patient.

Representative images of the chemotactic responses of wild-type *C. elegans* to 1 μl of a 10^{-1} dilution of urine from a control participant and a cancer patient. Urine was spotted at the '+' points, and 0.5 μl of 1 M sodium azide was spotted at the '+' and opposite points. The plates were photographed 1 h after the worms were placed at the start points (arrowheads).
