# Supplementary material for: A Highly Accurate Inclusive Cancer Screening Test Using Caenorhabditis elegans Scent Detection
Source: PLoS One. 2015 Mar 11;10(3):e0118699. doi: 10.1371/journal.pone.0118699 (PMC4356513; doi:10.1371/journal.pone.0118699)
Supplement: S6 Fig — Chemotactic responses of wild-type C. elegans to dilutions (10-0, 10-1, 10-2, 10-3 and 10-5) of urine samples from control participants (c1, c2 and c3) and cancer patients (p2, p5, p8, p17 and p18), n ≥ 5 assays. Background characteristics of participants are shown in S1 Table. (PDF) [file pone.0118699.s006.pdf]

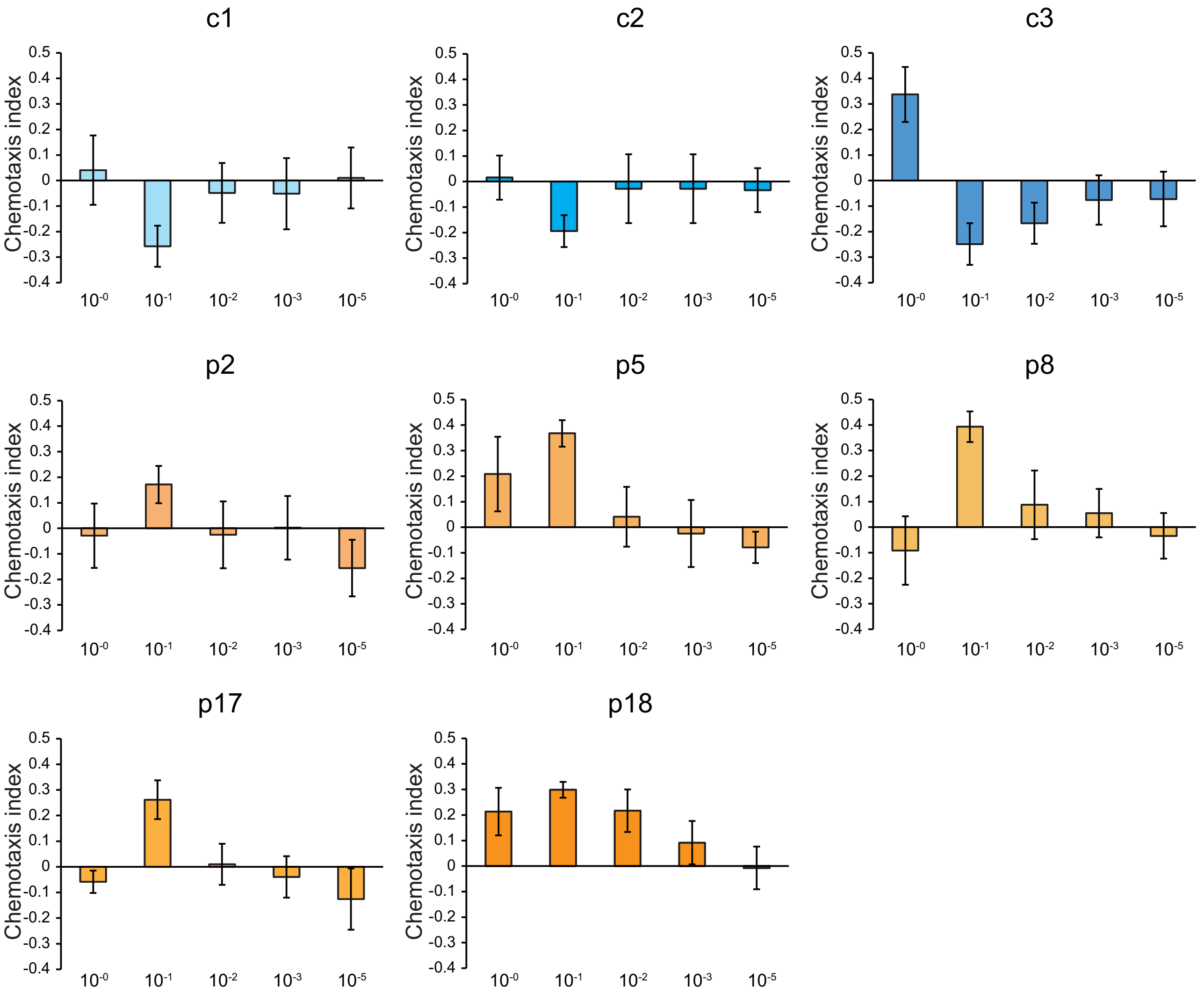

S6 Fig. Chemotaxis of wild-type *C. elegans* in response to various concentrations of urine from control participants and cancer patients.

Chemotactic responses of wild-type *C. elegans* to dilutions (10^{-0}, 10^{-1}, 10^{-2}, 10^{-3} and 10^{-5}) of urine samples from control participants (c1, c2 and c3) and cancer patients (p2, p5, p8, p17 and p18), $n \geq 5$ assays. Background characteristics of participants are shown in S1 Table.
