# Supplementary material for: A Highly Accurate Inclusive Cancer Screening Test Using Caenorhabditis elegans Scent Detection
Source: PLoS One. 2015 Mar 11;10(3):e0118699. doi: 10.1371/journal.pone.0118699 (PMC4356513; doi:10.1371/journal.pone.0118699)
Supplement: S7 Fig — (A) Chemotaxis of wild-type C. elegans in response to urine samples from control (E) or cancer patients (F, rectal cancer and G, sigmoid colon cancer), with or without filtration, which were used in the imaging experiments (n ≥ 5 assays). (B) Average fluorescence changes in AWC neurons for 10 s following urine removal (n ≥ 7 worms). Values are normalized to the average ratio change of Control-E. Error bars represent the SEM. Significant differences from the control are indicated by *** (P < 0.001), * (P < 0.05) as calculated by Student’s t-tests. (PDF) [file pone.0118699.s007.pdf]

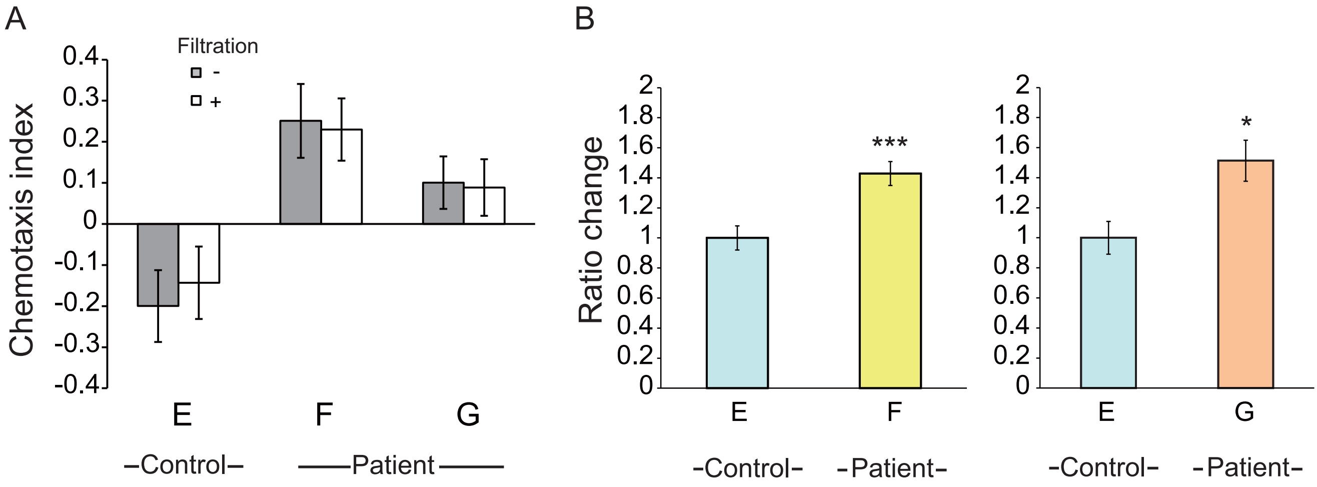

S7 Fig. Responses of AWC neurons to urine from patients with cancer.

(A) Chemotaxis of wild-type *C. elegans* in response to urine samples from control (E) or cancer patients (F, rectal cancer and G, sigmoid colon cancer), with or without filtration, which were used in the imaging experiments (n ≥ 5 assays). (B) Average fluorescence changes in AWCneurons for 10 s following urine removal (n ≥ 7 worms). Values are normalized to the average ratio change of Control-E. Error bars represent the SEM. Significant differences from the control are indicated by *** ($P < 0.001$), * ($P < 0.05$) as calculated by Student's *t*-tests.
