# Supplementary material for: A Highly Accurate Inclusive Cancer Screening Test Using Caenorhabditis elegans Scent Detection
Source: PLoS One. 2015 Mar 11;10(3):e0118699. doi: 10.1371/journal.pone.0118699 (PMC4356513; doi:10.1371/journal.pone.0118699)
Supplement: S1 Table — Sex, age, cancerous organ, cancer stage and CEA value for participants (10 controls and 20 cancer patients) are shown. M or F indicates male or female, respectively. A, D, S, and ca. indicates ascending, descending, sigmoid, and cancer, respectively. CEA indicates carcinoembryonic antigen. Cancer staging was based on Union Internationale Contre le Cancer (UICC) criteria. (PDF) [file pone.0118699.s009.pdf]

S1 Table. Limited background characteristics of participants**.**

		Gender	Age(years)	CEA (ng/ml)	Cancerous organ	Stage
Control	1	M	46	1.4	-	
	2	F	40	1.7	-	
	3	F	33	2.1	-	
	4	F	42	1.1	-	
	5	M	53	1.1	-	
	6	M	65	5.3	-	
	7	M	75	1.4	-	
	8	M	58	6.0	-	
	9	M	41	1.6	-	
	10	M	61	0.8	-	
Patient	1	M	62	1.1	Gastric ca.	I
	2	M	78	35.6	Gastric ca.	II
	3	F	64	0.9	Gastric ca.	I
	4	F	44	149.4	Rectal ca.	II
	5	M	73	69.8	Gastric ca.	II
	6	M	61	1.1	Gastric ca.	I
	7	F	84	3.3	D-colon ca.	II
	8	M	53	3.6	Gastric ca.	I
	9	M	46	2.9	Gastric ca.	II
	10	M	60	5.3	Gastric ca.	II
	11	M	87	4.0	S-colon ca.	I
	12	F	59	6.1	Gastric ca.	I
	13	M	58	42.6	Rectal ca.	II
	14	F	75	7.4	D-colon ca.	II
	15	M	79	3.0	Gastric ca.	II
	16	F	70	2.5	Pancreatic ca.	III
	17	M	73	3.7	A-colon ca.	IV
	18	M	58	27.3	Gastric ca.	IV
	19	M	81	3.7	Gastric ca.	II
	20	F	89	7.3	Gastric ca.	II

Sex, age, cancerous organ, cancer stage and CEA value for participants (10 controls and 20 cancer patients) are shown. M or F indicates male or female, respectively. A, D, S, and ca. indicates ascending, descending, sigmoid, and cancer, respectively. CEA indicates carcinoembryonicantigen. Cancer staging was based on Union Internationale Contrele Cancer (UICC) criteria.
