# Supplementary material for: A Highly Accurate Inclusive Cancer Screening Test Using Caenorhabditis elegans Scent Detection
Source: PLoS One. 2015 Mar 11;10(3):e0118699. doi: 10.1371/journal.pone.0118699 (PMC4356513; doi:10.1371/journal.pone.0118699)
Supplement: S2 Table — Data for sex, age, patient cancer history, number of patients with physical complaints and diseases other than cancer, and laboratory data are shown. NS, not significant. P values were calculated using the Student t test or chi-square test. WBC, Hgb, Plt, CRP, CEA, anti-p53 Ab and urine DiAcSpm/Cre indicate white blood cells, hemoglobins, blood platelets, C-reactive protein, carcinoembryonic antigen, anti-p53 antibody and urine N1, N12-diacetylspermine/creatinine, respectively. Certain complaints or other diseases indicate that the subject declared at least one complaint or disease other than cancer. Some TMs indicate a positive result for at least one of the tumour markers as follows: CEA, Anti-p53 Ab or DiAcSpm. (PDF) [file pone.0118699.s010.pdf]

S2 Table. Extended background characteristics of participants.

	Cancer		Control		Cancer	Control	*P*-value Cancer vs Control		NSDT(+)	NSDT(-)	*P*-value NSDT (+) vs (-)	
	2 years before	within 2 years	NSDT (+)	NSDT(-)								
n	19	5	11	207	24	218			34	208		
NSDT positive	19	5	11	0	24	11	5.90E-34	P<0.001				
Male Gender	12	3	2	87	14	89	0.10	NS	16	88	0.60	NS
Age (years)	47-89 (68)	53-70 (60)	41-73 (53)	26-78 (46)	47-89 (67)	26-78 (47)	7.90E-09	P<0.001	41-85 (65)	26-89 (46)	1.3E-08	P<0.001
Cancer history	0	0	0	6	0	6	0.90	NS	0	6	0.67	NS
Custom drinking	6	1	1	81	7	82	0.42	NS	8	81	0.07	NS
Smoked within 2 weeks	3	2	1	40	5	41	0.81	NS	6	40	0.76	NS
Complaints												
Cough or sputum	0	1	1	7	1	8	0.90	NS	2	7	0.85	NS
Appetite loss	1	1	0	0	2	0	0.002	P<0.01	1	1	0.65	NS
Abdominal distention	0	0	1	6	0	7	0.80	NS	1	6	0.60	NS
Weariness	1	1	2	15	2	17	0.76	NS	4	15	0.61	NS
Chest distention	1	1	1	4	2	5	0.30	NS	3	4	0.11	NS
Constipation or diarrhoea	3	1	0	9	4	9	0.04	P<0.05	4	9	0.19	NS
Headache	0	1	3	12	1	15	0.94	NS	4	12	0.38	NS
Bloody faeces	1	0	0	1	1	1	0.47	NS	1	1	0.67	NS
Pregnancy	0	0	0	3	0	3	0.69	NS	0	3	0.91	NS
Some complaints	9	2	5	48	11	53	0.02	P<0.05	15	49	0.01	P<0.05
Diseases other than cancer												
Hypertension	11	4	1	32	15	33	3.34E-08	P<0.001	15	33	1.2E-04	P<0.001
Hyperlipidemia	6	0	0	17	6	17	0.006	P<0.01	5	18	0.26	NS
Diabetes mellitus	3	0	0	12	3	12	0.18	NS	2	13	0.76	NS
Hyperuricemia	1	0	1	6	1	7	0.72	NS	1	7	0.69	NS
Ischemic heart disease	0	1	0	3	1	3	0.86	NS	1	3	0.91	NS
Cerebral infarction	2	0	0	0	2	0	0.002	P<0.01	1	1	0.65	NS
Collagen disease	0	0	0	1	0	1	0.18	NS	0	1	0.31	NS
Thyropathy	0	0	0	2	0	2	0.47	NS	0	2	0.67	NS
Bronchial asthuma	0	0	0	3	0	3	0.69	NS	0	3	0.91	NS
Gastro-duodenal ulcer	0	0	0	1	0	1	0.18	NS	0	1	0.31	NS
Chronic pancreatitis	0	0	0	1	0	1	0.18	NS	0	1	0.31	NS
Chronic hepatitis	0	0	1	4	0	5	1.00	NS	1	4	0.77	NS
Osteoarthritis	0	1	1	4	1	5	0.90	NS	2	4	0.46	NS
Uterine myoma	0	0	0	2	0	2	0.47	NS	0	2	0.67	NS
Mental disorder	0	0	0	1	0	1	0.18	NS	0	1	0.31	NS
Ophthalmologic disease	0	0	0	7	0	7	0.80	NS	0	7	0.58	NS
Some other diseases	15	4	5	73	19	78	3.85E-05	P<0.001	23	74	0.0004	P<0.001
Laboratory data												
WBC (×10^2 /μL) (median)	46.7-110.0 (55.0)	50.0-91.0 (57.0)	44.0-80.0 (56.0)	34.0-141.0 (60.0)	46.7-110.0 (56.0)	34.0-141.0 (60.0)	0.71	NS	44.0-110.0 (56.5)	34.0-141.0 (60.0)	0.51	NS
Hgb (g/dl) (median)	5.1-17.2 (13.9)	13.2-16.7 (14.6)	9.3-15.3 (13.1)	8.4-17.6 (14.1)	5.1-17.2 (14.1)	8.4-17.6 (14.1)	0.16	NS	5.1-17.2 (13.7)	8.4-17.6 (14.1)	0.37	NS
Plt (X10^4/μL) (median)	13.6-41.1 (20.5)	15.6-31.1 (18.5)	19.9-41.8 (24.2)	10.7-46.1 (23.2)	13.6-41.1 (19.75)	10.7-46.1 (23.25)	0.02	P<0.05	13.6-41.8 (21.9)	10.7-46.1 (23.2)	0.23	NS
Urine creatinine (mg/dl) (median)	0.32-1.63 (0.85)	0.25-0.77 (0.54)	0.10-1.26 (0.64)	0.12-2.56 (0.89)	0.25-1.63 (0.74)	0.10-2.56 (0.87)	0.22	NS	0.10-1.63 (0.74)	0.12-2.56 (0.88)	0.11	NS
CRP (>0.31mg/dl)	0.01-8.9 (0.08)	0.02-0.25 (0.17)	0.01-0.54 (0.04)	0.01-10.78 (0.04)	0.01-8.9 (0.105)	0.01-10.78 (0.04)	0.06	NS	0.01-8.9 (0.05)	0.01-10.78 (0.04)	0.20	NS
Tumor marker												
CEA (>5.0ng/ml)	5	1	0	8	6	8	2.16E-05	P<0.001	6	8	0.0019	P<0.01
Anti-p53 Ab (>1.30U/ml)	4	0	2	18	4	20	0.42	NS	6	18	0.12	NS
DiAcSpm/Cre Male>243,Female>354 (nmol/g･Cre)	4	0	0	10	4	10	0.39	NS	3	11	0.67	NS
Some TMs	10	1	2	34	11	36	0.00057	P<0.001	12	35	0.012	P<0.05

Data for sex, age, patient cancer history, number of patients with physical complaints and diseases other than cancer, and laboratory data are shown. NS, not significant. *P* values were calculated using the Student's *t*-test or chi-square test. WBC, Hgb, Plt, CRP, CEA, anti-p53 Ab and urine DiAcSpm/Cre indicate white blood cells, hemoglobins, blood platelets, C-reactive protein, carcinoembryonic antigen, anti-p53 antibody and urine N1,N12-diacetylspermine/creatinine, respectively. Certain complaints or other diseases indicate that the subject declared at least one complaint or disease other than cancer. Some TMs indicate a positive result for at least one of the tumour markers as follows: CEA, Anti-p53 Ab or DiAcSpm.
