# Supplementary material for: A Highly Accurate Inclusive Cancer Screening Test Using Caenorhabditis elegans Scent Detection
Source: PLoS One. 2015 Mar 11;10(3):e0118699. doi: 10.1371/journal.pone.0118699 (PMC4356513; doi:10.1371/journal.pone.0118699)
Supplement: S3 Table — The ORs and 95% confidence intervals (CIs) for cancer detection were estimated using five logistic regression models. Model 1: NSDT, age and complaints (appetite loss, constipation or diarrhoea, some complaints); Model 2: NSDT, age and other diseases (hypertension, hyperlipidaemia, cerebral infarction, some other diseases); Model 3: NSDT, age, Plt, CEA, anti-p53 Ab and DiAcSpm/Cre; Model 4: NSDT, age, hypertension, some other diseases, CEA and some positive TMs; Model 5: NSDT, age and CEA. The OR for each continuous variable was expressed as one standard deviation (SD) increase (0.20 for NSDT; 13.4 for age; 5.43 for Plt, 2.18 for CEA, 5.48 for anti-p53 Ab, 618 for DiAcSpm/Cre). Significant differences from control samples are indicated by * (P < 0.05), ** (P < 0.01), and *** (P < 0.001). (PDF) [file pone.0118699.s011.pdf]

S3 Table. Multivariate adjusted odds ratio for cancer detection

	One SD	Model 1		Model 2		Model 3		Model 4		Model 5	
		OR	95%CI	OR	95%CI	OR	95%CI	OR	95%CI	OR	95%CI
NSDT	0.20	48.3	6.30-370***	30.6	5.82-161***	49.8	5.75-432***	30.4	4.76-194***	32.6	5.35-198***
Age (years)	13.4	11.1	2.52-48.7**	7.38	1.93-28.1**	4.88	1.28-18.7*	9.36	2.17-40.3**	7.83	2.14-28.6**
Appetite loss		1.31	0.01-170								
Constipation or diarrhoea		222	0.38-0.29E+05								
Some complaints		10.7	0.96-119								
Hypertension				1.53	0.18-12.8			1.00	0.09-10.8		
Hyperlipidaemia				8.30	0.15-454						
Cerebral infarction				0.06	1.02E-90-3.97E+87						
Some other diseases				0.29	0.03-2.35			0.38	0.04-3.93		
Plt ($\times 10^4/\mu L$)	5.43					0.21	0.04-1.10				
CEA (ng/ml)	2.18					7.76	1.38-43.7*	3.87	0.91-16.5	4.07	1.18-14.0*
Anti-p53 Ab (U/ml)	5.48					3.43	0.34-34.8				
DiAcSpm/Cre (nmol/g･cre)	618					2.04	0.15-28.4				
Positive some tumor markers								0.10	0.08-14.7		

The ORs and 95% confidence intervals(CIs) for cancer detection were estimated using five logistic regression models. Model 1: NSDT, age and complaints (appetite loss, constipation or diarrhoea, some complaints); Model 2: NSDT, age and other diseases (hypertension, hyperlipidaemia, cerebral infarction, some other diseases); Model 3: NSDT, age, Plt, CEA, anti-p53 Ab and DiAcSpm/Cre; Model 4: NSDT, age, hypertension, some other diseases, CEA and some positive TMs; Model 5: NSDT, age and CEA. The OR for each continuous variable was expressed as one standard deviation (SD) increase (0.20 for NSDT; 13.4 for age; 5.43 for Plt, 2.18 for CEA, 5.48 for anti-p53 Ab, 618 for DiAcSpm/Cre). Significant differences from control samples are indicated by * ($P < 0.05$), ** ($P < 0.01$), and *** ($P < 0.001$).
